# Supplementary material for: Vaccine Hesitancy and Trust in the Scientific Community in Italy: Comparative Analysis from Two Recent Surveys
Source: Vaccines (Basel). 2021 Oct 19;9(10):1206. doi: 10.3390/vaccines9101206 (PMC8540200; doi:10.3390/vaccines9101206)
Supplement: Supplementary file 1 [file vaccines-09-01206-s001.zip › vaccines-1404686-supplementary.pdf]

Table S1

Descriptive characteristics of 2,626 respondents according to their opinion on harmfulness of vaccines (% in brackets). ESS Round 8.

		Vaccines are harmful				Missing
		**Agree**	**Undecided**	**Disagree**	**No answer**	**(n=0)**
	n = 2,626	n = 505 (19)	n = 535 (20)	n = 1302 (50)	n = 284 (11)	
Gender						0
Male	1284	249 (49)	264 (49)	619 (48)	152 (53)	
Female	1342	256 (51)	272 (51)	683 (52)	132 (47)	
Age classes						13
Less than 25	341	57 (11)	66 (12)	170 (13)	48 (17)	
25-44	752	153 (30)	167 (31)	372 (29)	61 (22)	
45-65	909	179 (36)	188 (36)	464 (36)	77 (27)	
65+	610	113 (23)	110 (21)	291 (22)	96 (34)	
Education (in years)						27
5	783	168 (34)	152 (29)	341 (26)	122 (44)	
8	1135	215 (43)	238 (45)	576 (45)	105 (38)	
13	348	67 (13)	77 (14)	178 (14)	25 (9)	
14+	334	49 (10)	62 (12)	200 (15)	23 (9)	
Religion						49
No	659	126 (25)	136 (26)	346 (27)	51 (19)	
Yes	1918	371 (75)	386 (74)	943 (73)	219 (81)	
Type of residence						3
Farm or home in countryside	97	16 (3)	19 (4)	44 (3)	17 (6)	
Country village	1149	201 (40)	275 (51)	551 (42)	121 (43)	
Town or small city	931	187 (37)	162 (30)	495 (38)	87 (30)	
Suburbs/ outskirts of a big city	156	34 (7)	31 (6)	75 (6)	16 (6)	
Big city	291	67 (13)	46 (9)	136 (11)	42 (15)	
Trust in scientific community						0
Agree strongly	446	35 (7)	28 (5)	375 (29)	7 (3)	

Agree	1111	178 (35)	187 (35)	689 (53)	57 (20)	
Neither agree nor disagree	584	139 (28)	269 (50)	146 (11)	30 (11)	
Disagree	171	85 (17)	33 (6)	44 (3)	9 (3)	
Disagree strongly	84	59 (12)	1 (0)	24 (2)	0 (0)	
Refusal	24	1 (0)	0 (0)	0 (0)	23 (8)	
Don't know	207	8 (2)	18 (3)	24 (2)	157 (55)	
Politics						26
Not at all interested	904	200 (40)	170 (32)	397 (31)	137 (50)	
Hardly interested	982	152 (31)	227 (43)	508 (39)	95 (34)	
Quite interested	600	127 (26)	120 (22)	314 (24)	39 (14)	
Very interested	115	17 (3)	16 (3)	77 (6)	5 (2)	
Political orientation						920
Right	489	111 (38)	102 (29)	243 (26)	32 (26)	
Centre	788	111 (37)	192 (54)	430 (46)	56 (45)	
Left	429	73 (25)	60 (17)	261 (28)	35 (29)	
State of health service in your country nowadays						31
0 (Very bad)	145	61 (12)	21 (4)	46 (4)	18 (6)	
1	79	32 (6)	15 (3)	29 (2)	4 (1)	
2	142	35 (7)	37 (7)	52 (4)	17 (6)	
3	212	48 (9)	40 (7)	94 (7)	30 (11)	
4	234	44 (9)	57 (11)	112 (9)	21 (8)	
5	353	54 (11)	82 (15)	183 (14)	34 (12)	
6	468	74 (15)	94 (18)	249 (19)	51 (19)	
7	491	73 (15)	101 (19)	265 (21)	51 (19)	
8	340	50 (10)	63 (12)	193 (15)	33 (12)	
9	84	20 (4)	14 (3)	41 (3)	9 (3)	
10 (Extremely good)	46	10 (2)	6 (1)	25 (2)	5 (2)	
Health status						11
Very bad	31	6 (1)	2 (0)	16 (1)	7 (3)	
Bad	121	24 (5)	21 (4)	56 (5)	20 (7)	

Fair	648	140 (28)	131 (25)	297 (23)	80 (29)	
Good	1192	229 (45)	257 (48)	602 (46)	104 (37)	
Very good	623	104 (21)	122 (23)	329 (25)	69 (24)	
Participation at congress, courses, training						23
No	327	44 (9)	481 (8)	218 (17)	22 (8)	
Yes	2276	458 (91)	481 (92)	1079 (83)	258 (92)	
How often do you use Internet						0
Never	592	123 (24)	106 (20)	253 (19)	109 (39)	
Only occasionally	184	44 (9)	51 (9)	74 (6)	15 (5)	
A few times a week	179	33 (7)	42 (8)	85 (7)	20 (7)	
Most days	388	77 (15)	89 (17)	184 (14)	37 (13)	
Every day	1267	222 (44)	240 (45)	704 (54)	100 (35)	
Don't know	16	6 (1)	7 (1)	1 (0)	2 (1)	
Most people can be trusted or you can't be too careful						0
0 (You can't be too careful)	224	56 (11)	22 (4)	101 (8)	45 (16)	
1	117	29 (6)	17 (3)	62 (5)	9 (3)	
2	207	36 (7)	44 (8)	104 (8)	23 (8)	
3	249	57 (11)	53 (10)	111 (9)	28 (10)	
4	301	61 (12)	72 (13)	137 (10)	32 (11)	
5	553	81 (16)	134 (25)	280 (21)	58 (20)	
6	405	83 (16)	89 (17)	190 (15)	43 (15)	
7	320	52 (10)	54 (10)	186 (14)	27 (10)	
8	189	32 (6)	37 (7)	105 (8)	15 (5)	
9	28	7 (2)	6 (1)	12 (1)	2 (1)	
10 (Most people can be trusted)	27	9 (2)	5 (1)	12 (1)	1 (1)	
Refusal	1	0 (0)	1 (0)	0 (0)	0 (0)	
Don't know	6	2 (1)	2 (1)	1 (0)	1 (0)	

Table S2.

Descriptive characteristics of 2,745 respondents according to their opinion on harmfulness of vaccines (% in brackets). ESS - Round 9.

		Vaccines are harmful				
		Agree	Undecided	Disagree	No answer	Missing
	n = 2,745	n = 381 (14)	n = 412 (15)	n = 1740 (63)	n = 212 (8)	
Gender						0
Male	1297	186 (49)	205 (49)	804 (46)	102 (49)	
Female	1448	196 (51)	210 (51)	935 (54)	106 (51)	
Age classes						21
Less than 25	316	38 (10)	54 (13)	189 (11)	35 (17)	
25-44	703	94 (25)	122 (30)	440 (25)	47 (23)	
45-65	918	147 (39)	142 (35)	585 (34)	44 (21)	
65+	787	99 (26)	91 (22)	517 (30)	80 (39)	
Education (in years)						85
0-7	363	42 (11)	49 (12)	215 (12)	57 (28)	
8-12	1009	171 (45)	159 (39)	596 (34)	84 (41)	
13	609	79 (21)	101 (25)	407 (24)	22 (11)	
14+	680	68 (18)	89 (22)	494 (29)	29 (14)	
Religion						41
No	566	80 (21)	103 (25)	347 (20)	36 (18)	
Yes	2138	294 (79)	304 (75)	1379 (80)	161 (82)	
Type of residence						9
Farm or home in countryside	95	12 (3)	14 (3)	60 (3)	10 (5)	
Country village	1179	182 (48)	183 (44)	715 (41)	98 (48)	
Town or small city	956	113 (30)	135 (32)	648 (37)	60 (29)	
Suburbs/ outskirts of a big city	168	20 (5)	33 (8)	99 (6)	15 (7)	
Big city	338	50 (13)	50 (12)	216 (12)	22 (11)	
Trust in scientific community						
Agree strongly	737	57 (15)	27 (7)	647 (37)	6 (3)	
Agree	1174	116 (30)	121 (29)	895 (51)	43 (20)	

Neither agree nor disagree	453	74 (19)	241 (58)	122 (7)	16 (8)	
Disagree	158	93 (24)	18 (4)	40 (2)	6 (3)	
Disagree strongly	71	39 (10)	3 (1)	24 (1)	5 (2)	
Refusal	16	1 (0)	1 (0)	0 (0)	14 (7)	
Don't know	137	3 (1)	3 (1)	12 (1)	119 (57)	
Politics						8
Not at all interested	863	137 (36)	147 (36)	469 (27)	109 (53)	
Hardly interested	1046	145 (38)	162 (39)	667 (39)	73 (35)	
Quite interested	679	82 (22)	88 (21)	487 (28)	23 (11)	
Very interested	149	14 (4)	18 (4)	114 (7)	3 (1)	
Political orientation						744
Right	620	97 (34)	84 (28)	411 (31)	28 (29)	
Centre	935	136 (48)	160 (54)	588 (44)	51 (52)	
Left	446	49 (17)	51 (17)	327 (25)	19 (19)	
State of health service in your country nowadays						32
0 (Very bad)	97	20 (5)	10 (2)	55 (3)	12 (6)	
1	61	10 (3)	7 (2)	38 (2)	6 (3)	
2	120	24 (7)	21 (5)	67 (4)	7 (4)	
3	185	29 (8)	26 (6)	118 (7)	12 (6)	
4	220	36 (10)	43 (11)	125 (7)	15 (8)	
5	358	58 (15)	55 (13)	215 (12)	30 (15)	
6	453	57 (15)	75 (18)	293 (17)	28 (14)	
7	579	59 (16)	98 (24)	386 (22)	36 (18)	
8	453	52 (14)	55 (13)	316 (18)	31 (16)	
9	130	24 (6)	13 (3)	84 (5)	9 (5)	
10 (Extremely good)	56	5 (1)	8 (2)	32 (2)	10 (5)	
Health status						9
Very bad	35	4 (1)	6 (1)	18 (1)	7 (3)	
Bad	134	23 (6)	17 (4)	70 (4)	24 (12)	

Fair	716	124 (33)	94 (23)	442 (25)	56 (27)	
Good	1241	152 (40)	199 (48)	815 (47)	76 (37)	
Very good	610	79 (21)	99 (24)	392 (23)	41 (20)	
Participation at congress, courses, training						21
No	2409	350 (94)	375 (91)	1493 (86)	192 (95)	
Yes	315	21 (6)	38 (9)	244 (14)	11 (5)	
How often do you use Internet						5
Never	597	88 (23)	85 (20)	348 (20)	77 (37)	
Only occasionally	211	44 (12)	34 (8)	120 (7)	13 (6)	
A few times a week	190	23 (6)	33 (8)	122 (7)	12 (6)	
Most days	361	63 (17)	55 (13)	217 (12)	25 (12)	
Every day	1381	161 (43)	208 (50)	931 (54)	82 (40)	
Don't know						
Most people can be trusted or you can't be too careful						9
0 (You can't be too careful)	187	27 (7)	38 (9)	98 (6)	23 (12)	
1	105	17 (5)	13 (3)	62 (4)	13 (7)	
2	225	30 (8)	37 (9)	131 (8)	27 (14)	
3	296	39 (10)	54 (13)	182 (11)	21 (11)	
4	279	31 (8)	29 (7)	202 (12)	17 (9)	
5	498	84 (22)	76 (18)	304 (18)	34 (18)	
6	408	49 (13)	69 (17)	264 (15)	26 (13)	
7	432	60 (16)	51 (12)	293 (17)	27 (14)	
8	231	32 (9)	36 (9)	150 (9)	12 (6)	
9	37	6 (2)	4 (1)	23 (1)	4 (2)	
10 (Most people can be trusted)	40	4 (1)	6 (1)	28 (2)	1 (1)	
Refusal	1	0 (0)	1 (0)	0 (0)	0 (0)	
Don't know	8	2 (1)	3 (1)	0 (0)	3 (2)	
